# The “cost” of treating to target: cross-sectional analysis of patients with poorly controlled type 2 diabetes in Australian general practice

**DOI:** 10.1186/1471-2296-14-32

**Published:** 2013-03-08

**Authors:** John Furler, Justin W S Hii, Danny Liew, Irene Blackberry, James Best, Leonie Segal, Doris Young

**Affiliations:** 1 Department of General Practice, The University of Melbourne, Melbourne, Australia; 2 Melbourne EpiCentre, Royal Melbourne Hospital, The University of Melbourne, Melbourne, Australia; 3 Department of Medicine, St Vincent’s Hospital, The University of Melbourne, Melbourne, Australia; 4 Division of Health Sciences, University of South Australia, Adelaide, Australia

**Keywords:** Type 2 diabetes, Cost, Treatment gap, Treatment burden

## Abstract

**Background:**

To describe the current treatment gap in management of cardiovascular risk factors in patients with poorly controlled type 2 diabetes in general practice as well as the associated financial and therapeutic burden of pharmacological treatment.

**Methods:**

Cross-sectional analysis of data from the Patient Engagement and Coaching for Health trial. This totalled 473 patients from 59 general practices with participants eligible if they had HbA1c > 7.5%. Main outcome measures included proportions of patients not within target risk factor levels and weighted average mean annual cost for cardiometabolic medications and factors associated with costs. Medication costs were derived from the Australian Pharmaceutical Benefits Schedule.

**Results:**

Average age was 63 (range 27-89). Average HbA1c was 8.1% and average duration of diabetes was 10 years. 35% of patients had at least one micro or macrovascular complication and patients were taking a mean of 4 cardio-metabolic medications. The majority of participants on treatment for cardiovascular risk factors were not achieving clinical targets, with 74% and 75% of patients out of target range for blood pressure and lipids respectively. A significant proportion of those not meeting clinical targets were not on treatment at all. The weighted mean annual cost for cardiometabolic medications was AUD$1384.20 per patient (2006-07). Independent factors associated with cost included age, duration of diabetes, history of acute myocardial infarction, proteinuria, increased waist circumference and depression.

**Conclusions:**

Treatment rates for cardiovascular risk factors in patients with type 2 diabetes in our participants are higher than those identified in earlier studies. However, rates of achieving target levels remain low despite the large ‘pill burden’ and substantial associated fiscal costs to individuals and the community. The complexities of balancing the overall benefits of treatment intensification against potential disadvantages for patients and health care systems in primary care warrants further investigation.

## Background

Australia, like the rest of the world, is in the midst of an epidemic of diabetes. Over a million Australians have diabetes, of which over 85% is type 2 (T2DM) [1]. The prevalence more than doubled from 1989–90 to 2004–05 and is predicted to continue rising. Diabetes and its complications contributed 8.3% of the total burden of disease in Australia in 2003. It shortens life expectancy by up to 5 years and costs the community nearly $1 billion annually in direct health care costs [2], a figure that is expected to treble over the next 40 years [3]. Future scenarios suggest a progressive rise in the human and health care costs associated with diabetes, particularly related to cardiovascular disease and the increasingly identified co-morbid depression.

Evidence-based clinical care focused on treating to targets for risk factors can improve outcomes for people with T2DM [4]. While patient-centred education and self-management interventions are important [5,6], pharmacotherapy is a key element of treatment for T2DM, both for glycaemic control and associated risk factors such as dyslipidaemia and hypertension. Targeting treatment to high risk patients is felt to be important [7].

In Australia, in line with other developed countries, there has been significant investment in programs to improve quality of clinical care and outcomes for diabetes. Over the last two decades, these have included the National Divisions Diabetes Program [8], the Australian Primary Care Collaboratives [9], the National Integrated Diabetes Program and targeted incentive payments to GPs and practices [10]. The Diabetes Care Project is currently underway, to trial a system of voluntary capitation payments to fund care for patients with diabetes in general practice, with the aim of improving comprehensive high quality care and reducing downstream costs (http://www.dcp.org.au).

A decade ago, early in the life of these initiatives, the gap between treatment goals and their translation into clinical practice remained wide. An Australian study reporting 2002 data suggested that patients with known diabetes identified in Australian general practices had poorly controlled disease and associated cardiovascular risk factors [11]. That study identified 48%, 88% and 74% of patients out of target for HbA1C, cholesterol levels and blood pressure (BP), respectively. The authors concluded that GPs needed to be more active, particularly in targeting treatment to patients with higher risk. While targets vary from country to country, these results are broadly comparable to those reported in the UK and US at that time, both in published studies and in analysis of national Quality and Outcomes Framework (QOF) data [12,13].

We report here more recent cross-sectional data from the Patient Engagement and Coaching for Health (PEACH) study, a cluster randomised controlled trial of practice nurse-led telephone coaching for patients with poorly controlled T2DM in Australian general practices [14]. Our primary objective is to describe the treatment gap in management of cardiovascular risk factors, both in the initiation, as well as the intensification, of treatment to achieve targets. Our secondary objective is to describe the significant ‘pill burden’ borne by patients along with the associated financial cost and factors associated with this cost.

## Methods

The PEACH study has been described elsewhere [14]. Practices were recruited from across Victoria via mail-outs and newsletter promotions through Divisions of General Practice, and patients were identified from practice electronic databases or local pathology services. Patients with type 2 diabetes were eligible if their most recent HbA1c was within the last 12 months and was greater than 7.5%. Patients were recruited sequentially over 18 months through to August 2008. Baseline data for each patient was collected within 4 weeks of recruitment.

Practice nurses (PNs) collected data via face-to-face interviews. Height and weight were collected by the PN according to standard instructions. BP was recorded using usual clinical practice. The nurses also extracted data from the patients’ medical records, including information on complications and current medication use. Data on medication use was based on what was prescribed by the GP and was recorded in medical record at the practice. PNs were asked to confirm drug usage on the list and also check for any additional complimentary medications taken. The PN then arranged baseline pathology testing (HbA1c, lipids, renal function and urinalysis). All the pathology laboratories used HbA1c assay methods aligned with DCCT [15] and undertook quality assurance for HbA1c and other biochemical assays.

The PEACH study adopted target levels for risk factors from the diabetes clinical guidelines in use in general practice at the time [16] and from the National Heart Foundation (Table 1).

**Table 1:** Targets for telephone coaching

**Clinical measure**	**Target**
HbA1c	< 6.5%
Total cholesterol	<4.0 mmol/L
LDL cholesterol	<2.0 mmol/L
BP if microalbuminuria absent	< 130/80
BP if microalbuminuria present	< 125/75
Renal protection	All patients with microalbuminuria to be on angiotensin converting enzyme inhibitor (ACEi) or angiotensin II receptor blocker (ARB) unless contraindicated
Anti-thrombotic	All patients to be on anti-platelet agent unless contraindicated

**Figure 1:**
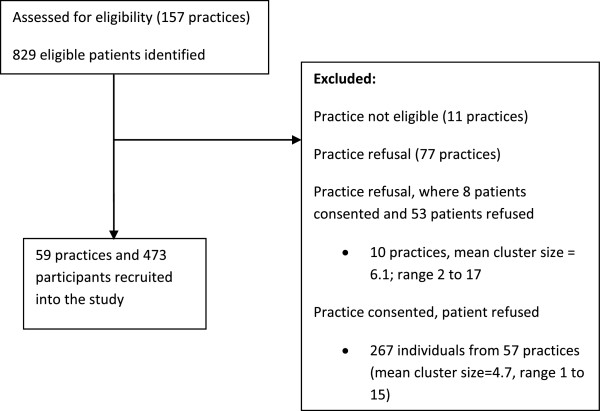
Flowchart of participant recruitment.

The primary outcome of the PEACH trial was HbA1c level at 18 months post baseline. For 80% power and 5% significance level (two-sided test), 464 patients (8 patients per practice) from 58 primary care practices were required (232 per group) to detect an absolute 0.5% reduction in mean HbA1c between the intervention and control groups. Sample size was based on a two-sample t-test assuming a standard deviation of 1.44 and was inflated by 1.3 to allow for the correlation of the patient outcomes belonging to the same practice (assuming an intra-cluster correlation of 0.05 and variable sample cluster sizes) and allows for a 20% attrition rate over 18 months. A flow chart of patient recruitment is shown in Figure 1.

The PEACH study was funded by the National Health and Medical Research Council (NHMRC) General Practice Clinical Research Grants program. Ethics approval was granted by the University of Melbourne Human Research Ethics Committee. The trial is registered with Current Controlled Trials (ISRCTN 50662837).

### Medication cost estimation

Medications were classified into the following groups: cardiovascular (antihypertensive, antiarrhythymic, antianginal, lipid lowering, inotropic, anticoagulants and antiplatelets), diabetic (oral hypoglycaemics and insulin), antidepressants and ‘other’. Cardiometabolic medications encompassed both cardiovascular and diabetic medications. The use of antidepressant medications was used as a surrogate marker for the presence of depression. We estimated the costs of cardiovascular, diabetic and antidepressant medications only.

Medication costs were based on reimbursed prices in the Australian Pharmaceutical Benefits Schedule (PBS) in 2006–7. These were the costs met by the Australian Government which funds 84% of the costs of benefit-paid pharmaceuticals in Australia, (and 52% of the costs of all medications). They do not include co-payments by consumers. The estimation of costs was based on the method adopted in another Australian study [17]. In summary, for each therapeutic class, a weighted average daily cost was estimated by calculating the sum-product of the cost of each drug dose and its proportional contribution to the class based on the market share as listed on the PBS. For example, assume that two drugs existed in a class and cost $100 and $200 per year, and comprised 60% and 40% of the market share of the class, respectively. The weighted average cost of that class would be $100*60% + $200*40% = $140 per year. The various doses of the drug, costs and services supplied were sourced from the PBS based on the period September 2006–Sept 2007 [18]. Where a particular drug was available in different doses, we assumed that the doses used were those recommended in treatment guidelines [19-22]. The underlying assumption with the above method for cost estimation is that patients were fully compliant with their medications.

### Statistical analyses

Descriptive analyses were undertaken on the baseline patient data from the PEACH study.

Statistical analyses for assessing treatment gap were performed using SPSS for Windows version 19.0 (IBM Corp., New York, NY). To determine proportions of patients not within target risk factor levels, risk factor prevalence data were cross-tabulated against data on relevant medication use. Statistical analyses for the costing of medications were performed using PASW for Mac version 18.0. First, a series of univariate linear regression analyses were undertaken to identify significant factors associated with cardiovascular and diabetes (cardio-metabolic) medication costs. Significant variables from these analyses were then entered into a multivariate linear regression model to identify independent factors associated with cardio-metabolic medication costs. P values of less than 0.05 were considered statistically significant.

## Results

The baseline characteristics of participants are summarised in Tables 2 (demographic) and 3 (clinical). Age ranged from 27-89 years. 13 patients had a diabetes duration of less than 1 year recorded. Over one third (34%) of participants had a record in the medical file of at least one diabetes complication (micro and/or macrovascular).

**Table 2:** Demographics of participants (n = 473)

	
Age (years) Mean (SD)	63 (11)
Sex (%) Male	57
Smoker (%)	12
Current	
Highest Level of Education (%)	
Primary	22
Completed Secondary School	42
Trade or TAFE	19
Completed University Degree	17
Employment Status (%)	
Employed	34
Unemployed	8
Home Duties	7
Retired	48
Health Care Card (%)	62

**Table 3:** Clinical characteristics of participants

**Diabetic characteristics**	**Mean (SD)**	**Median**	**Range**	**Inter-quartile Range**
HbA1c (%)	8.1 (1.3)	7.8	5.3 – 16.0	7.3 – 8.6
Diabetes Duration (years)	10 (7)	10	0 – 44	5 – 14
Systolic BP (mmHg)	138 (18)	138	90 – 221	126 – 149
Diastolic BP (mmHg)	79 (10)	80	53 – 115	72 – 85
Total Cholesterol (mmol/L)	4.5 (1.1)	4.4	2.5 – 12.4	3.7 – 5.0
Waist Circumference (cm)	109 (15)	108	68 – 167	99 – 118
BMI (kg/m^2^)	32.8 (6.5)	31.7	19.8 – 54.5	28.1 – 36.2
Medications (total)	7.5 (4.1)	7	1 – 24	5 – 10
Medications (Cardio-metabolic)	4.2 (2.2)	4	0 – 13	3 – 5
**Diabetes complications**	**Rate**			
Any	34.1% (155/434)			
Macro				
AMI	16.8%			
CVA	5.5%			
PVD	2.5%			
Micro				
Retinopathy	20.7%			
Neuropathy	6.6%			
Nephropathy	12.4%			

The mean number of cardiometabolic medications were 4.2 (SD 2.2) with a range of 0–13. The mean number of total medications were 7.5 (SD 4.1) with a range of 1–24. 33.2% of participants were prescribed antidepressant medications at antidepressant doses. The proportions of medications are shown in Table 4.

**Table 4:** Proportions of cardiometabolic medications

**Medication**	**Frequency**
Hypoglycaemic	
Insulin	23% (109/473)
Metformin	76.7% (415/473)
Other oral Hypoglycaemics	87.7% (415/473)
Hypolipidaemic	65.1% (308/473)
Statins	62.8% (297/473)
Fibrate	2.7% (13/473)
Ezetimibe	4.2% (20/473)
Antihypertensives	71.5% (338/473)
Beta blocker	18.6% (88/473)
ACEi	40.2% (190/473)
ARB	33.8% (160/473)
Antiplatelets	46.9% (222/473)
Aspirin	44.2% (209/473)
Dipyridamole	1.3% (6/473)
Clopidogrel	4.9% (23/473)
Warfari	5.9% (28/473

Table 5 shows the prevalence of cardiovascular risk factors. Our sample had high prevalence of hypertension and dyslipidaemia, with 74% and 75% being out of target range for BP and lipids, respectively. Significant proportions of participants with a risk factor were not on treatment (see right hand column of Table 5). Most participants (73%) with albuminuria were taking angiotensin converting enzyme inhibitors (ACEi) and/or angiotensin receptor blockers (ARB). Lastly, only 47% of the sample was taking anti-platelet agents.

**Table 5:** Risk factor prevalence and treatment

**Risk factor**	Prevalence n/N (%)*	**Number (%) of patients with risk factor not on treatment**
BP out of target range	341/460 (74)	86 (25)
Lipids out of target range	319/424 (75)	128 (40)
Microalbuminuria or albuminuria present	122/428 (29)	35 (27) (ACEi or ARB)
Thrombotic risk present (assumed to be all participants)	473/473 (100)	250 (53) (antiplatelet)

* Risk factor prevalences have differing denominators due to some missing data.

**Figure 2:**
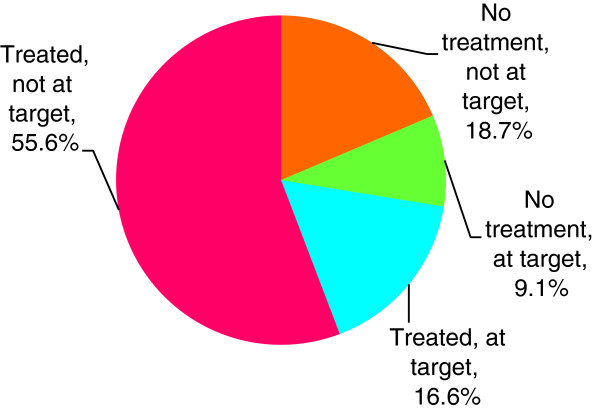
Blood pressure (n = 460).

**Figure 3:**
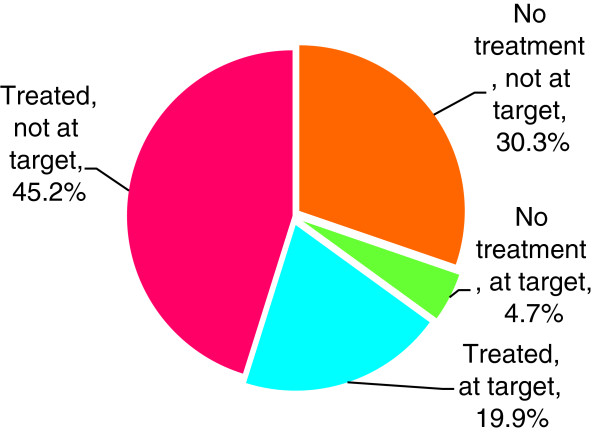
Lipids (n = 424).

Figures 2 and 3 illustrate the overall distribution of patients achieving target and on treatment for BP and lipids. Among patients who were on treatment for a risk factor, the majority remained out of target: 76% of patients on antihypertensive treatment and 70% of those on lipid-lowering treatment.

The mean average weighed cost per year for these medications was AUD$1384 (SD $850). Increasing age, greater duration of diabetes, a history of acute myocardial infarction (AMI), depression and the presence of proteinuria were independent factors associated with cardio-metabolic medication costs (Table 6).

**Table 6:** Weighted average mean and independent predictors of cardiometabolic medication costs

**Variable**	**Mean cost variance ($AU)**	**p value**	**95% CI**
AMI	+ 1,018.45	0.000	542.88 – 1494.02
Depression	+ 486.45	0.000	320.73 – 652.16
Proteinuria	+ 215.10	0.018	36.76 – 393.44
Duration of diabetes (+ 1 year)	+ 18.43	0.001	7.29 – 29.57
Age (+ 1 year)	+ 8.18	0.040	0.36 – 16.00
Waist Circumference	+ 7.45	0.005	2.21 – 12.69

Costs were significantly lower for patients without any recorded diabetes complications (Table 7).

**Table 7:** Cost of cardiometabolic medications for those with and without complications

**Complication**	**N**	**Mean (AUD)**	**SD**
None	307	1235.80	811.80
Any	166	1658.67	851.65

p value for difference <0.001.

## Discussion

Cardiovascular disease is the main cause of death for people with T2DM, but with multi-factorial clinical interventions, significant improvements in absolute cardiovascular risk can be achieved [7]. Our primary objective was to describe the treatment gap in management of cardiovascular risk factors, both in the initiation, as well as the intensification of treatment to achieve targets. Our findings identify that among patients with poorly controlled T2DM in Australian general practice, the majority are on treatment for associated cardiovascular risk factors.

We identified higher rates of treatment compared to a decade ago in Australia [11]. This improvement may represent the influence of guidelines and incentive schemes and initiatives, although it may also represent selection of a group with more severe T2DM, as our patients were selected with HbA1c above 7.5%. However, despite the higher rates of treatment, our data shows that substantial numbers remain untreated, contrary to evidence-based guidelines. Our study also identified that the majority of treated patients were not achieving targets for BP and lipids, a finding in accord with earlier studies [11,23]. Similar findings were identified in the UK [12] where rates of being “in-target” for BP and cholesterol amongst patients with type 2 diabetes were 36–70% and 41–72% respectively (although targets used in these studies were higher than those used in the current study). Similar findings were identified in the US [13,24].

Our secondary objective was to describe the number of medications participants were taking together with estimated costs associated with pharmacological treatment from the perspective of the Australian government. We found a high “pill burden” amongst our participants. While there is international population level data about trends to increasingly intense, complex and costly treatment regimes for diabetes [25,26], there is very little data published on the medication burden of individual patients. A study of 128 patients in the US [27] found they were taking a mean of 4.1 medications to control diabetes and an overall mean pill burden of 5.8 different medications. A study of 1,991 US primary care patients with a HbA1c of 9.1 +/− 1.3 found they were taking a mean of 9.1 medications in total [28]. Australian data suggests that amongst all people with diabetes less than 1% use four or more cardiometabolic medications whereas this was the average figure in our study [29].

Pharmaceuticals comprise one of the fastest growing costs within the health care systems. In Australia medications for the treatment of cardiovascular disease and diabetes are significant contributors to total PBS cost [18]. Given the rapidly rising prevalence of T2D and continued poor glycaemic control, the estimated mean average weighted cost per year for cardiometabolic medications of more than AUD$1384 (SD $850) per individual represents a considerable encumbrance on the PBS system. Predictably, a history of AMI was the most significant independent factor associated with pharmaceutical cost, increasing costs by over AUD$1000 annually. Any complication added significantly to medication costs. Importantly, depression also independently contributed to costs of medications, with the excess cost associated being almost half that of AMI ($AUD486 annually). Part of the reason for this increased cost was that patients with depression used more cardio-metabolic medications (mean 5.4 vs 3.6, p <0.0001).

In the context of two decades of initiatives and incentive schemes aimed at improving diabetes outcomes, is this apparent failure on the part of GPs and patients to embrace more intensive pharmacological treatment for T2D simply another example of clinical inertia [28,30,31]? From a clinical perspective, this might suggest that new strategies such as care coordinators or coaches [32] are needed to drive further intensification of treatment. After all, if patients are to be prescribed medications, they ought reasonably to expect clinical benefit from them. Our findings may also suggest the need for renewed effort in detecting and managing depression in people with diabetes. We need open discussion of the cost of such measures and the implications for sustainability of the health system.

However, our findings may also reflect collective views of GPs and patients about the primary aim of care for the patient with diabetes. There is debate about the diminishing clinical benefit and increasing personal and lifestyle costs associated with the relentless pursuit of surrogate markers in diabetes and cardiovascular disease to the exclusion of hard endpoints that matter to patients [33,34]. Similar issues have been raised in relation to the pharmacological management of depression [35]. GPs may be sensitive to this issue in their close relationships with patients, aware of the burden of treatment that patients bear and are reluctant to add to it [36]. Our findings may reflect decisions made by GPs and their patients to limit their pursuit of biological markers at the cost of quality of life and other patient-centred outcomes. This may reflect an attempt by GP and patient to say “*enough is enough*”, to tailor clinical decisions to the personal context of the patient. This attention to the way treatment can add complexity and “work” to the daily lives and circumstances of patients living with chronic conditions is an attempt to recast the issue of “non-compliance”. Practitioners and patients in primary care may both be implicitly engaging in what has been called “*minimally disruptive medicine*” [37], balancing what is realistic and achievable for patients against evidence based guidelines driven by disease markers.

Understanding how patients and health practitioners engage in this work, how they interpret evidence and guidelines while incorporating outcomes of importance to patients warrants much more investigation. Development of robust measures of patient-centred outcomes to allow their incorporation into guidelines in ways that account for complexity, multiple morbidity and social context, is a key area of research if evidence is to be relevant to the realities of practice [38].

One important limitation of our study is selection bias. Patients were recruited into the study on the basis of poor glycaemic control. This may have led to a sample of patients with poorer control of other cardiovascular risk factors. Our results cannot be transferred to type 2 diabetes patients in general, but are only interpretable for this selected group. Another potential limitation is the reliability of documentation of complications in the participants’ medical records. Complication rates were determined by the PN from the medical record based on recorded diagnoses. There may be significant variation between practices in the documentation of individual complication rates. We were also unable to perform quality checks on the data extraction by PNs. This may have led to underreporting of complications. Because we assumed no contraindications to treatment, the extent of under-treatment is likely to have been over-estimated. Our use of antidepressant medications at antidepressant doses as a surrogate marker for depression may be an overestimate as some patients may be on treatment for other conditions such as anxiety. On the other hand, we did not count those with depression but not on pharmacotherapy. Our costing was estimated and prices may vary between non-concessional patients. In addition, it is very difficult to estimate costs due to private pharmacy costs, which can vary enormously. However, as 62% of our patients were receiving health care card benefits, our costing was likely to have been an underestimate. Finally, the underlying assumption underpinning annual costs was that patients remained compliant with their medication throughout the year. Despite its limitations, participation of a large sample of primary care patients across Victoria is a major strength of our study, as is the collection of detailed patients’ medications data.

## Conclusion

Despite significant investment of money and resources at system and practice level, and modest improvement in treatment rates for cardiovascular risk factors in patients with type 2 diabetes in Australian general practice, there is still opportunity to intensify treatment to achieve target levels. We did find evidence that greater intensity of treatment is directed to those with higher risk. More patients are on now treatment and for the majority of patients, the pill burden continues to rise, at significant cost to funders, while improved disease markers and outcomes remain difficult to achieve. The challenge is to understand better how practitioners and patients balance evidence with the complex realities of practice and to develop and include measures within guidelines that reflect important patient priorities.

## Competing interests

The authors declare that they have no competing interests.

## Authors’ contributions

DY and JB conceptualised the research. DY, JB, JF and LS conceived the development of study design. DY, IB and JF oversaw the running of the trial. JF, JH, DL drafted the manuscript. JH, DL, JF and IB conducted the data analysis. All authors read and approved the final manuscript.

## Pre-publication history

The pre-publication history for this paper can be accessed here:

http://www.biomedcentral.com/1471-2296/14/32/prepub
